# Addiction to RUNX in lymphoma

**DOI:** 10.18632/aging.101071

**Published:** 2016-09-28

**Authors:** James C. Neil, Gillian Borland, Anna Kilbey

**Affiliations:** Molecular Oncology Laboratory, Centre for Virus Research, Institute of Infection, Immunity and Inflammation, University of Glasgow, Bearsden Glasgow G61 1QH, UK

**Keywords:** RUNX, oncogene addiction, lymphoma, Myc, p53

The three mammalian *Runx* genes encode transcription factors that play essential but distinct and lineage-specific roles in development. These sequence-specific DNA binding proteins share a common binding cofactor (CBFβ) that confers protein stability and high affinity for target DNA on its RUNX partners. An important link to cancer was first realised through identification of both *RUNX1* and *CBFB* as frequent targets for chromosomal translocations in human leukaemia. Early studies suggested that RUNX1 is a tumour suppressor subject to dominant negative inhibition by its fusion oncoprotein derivatives and to loss-of-function mutations in AML (reviewed in [1]). However, it is now clear that RUNX1 is far from a typical tumour suppressor as, for example, AML cells expressing the RUNX1-ETO fusion require the activity of the unaffected allele for survival [2] while ALL cases frequently over-express *RUNX1* and/or display increased copy number [1]. Moreover, early studies on mouse models of lymphoma revealed all three *Runx* genes as targets for transcriptional activation in MYC transgenic mice, and the ability of over-expressed MYC and Runx to synergise in lymphoma has been amply confirmed in compound transgenics [1].

Our recent study [3] sheds further light on the dualistic behaviour of the *RUNX* genes and validates their basal activities as potential targets for therapeutic intervention. By introducing a conditional knockout allele of *Runx1* into the well-established Eμ-Myc model we showed that primary lymphoma cells strongly select for retention of both wild-type alleles while normal splenic lymphocytes can survive monoallelic deletion. Notably, normal myeloid cells are permissive for full deletion of *Runx1*, which may in part account for its preferential tumour suppressor activity in the myeloid lineage [1]. In contrast to primary Eμ-Myc lymphomas, established cell lines which have lost p53 survive complete deletion of *Runx1*. However, deficiency is not without cost, as *Runx1^null^* cells proliferate more slowly and display increased sensitivity to the cytotoxic effects of glucocorticoids and DNA damage. Transcriptome analysis is consistent with this phenotype, as significantly altered probes were over-represented for genes controlling B-cell proliferation, survival and differentiation. Intriguingly, *Rag1* and *Rag2* were among the most strongly de-repressed genes after Runx1 deletion, providing a mechanistic rationale for the frequent occurrence of RAG-induced mutations in t(12;21) leukemias where TEL-RUNX1 compromises RUNX functions [4].

At first sight our findings contrast with a recent report that Runx1 deficiency in normal haematopoietic progenitors leads to reduced cell size due to down-regulation of genes involved in ribosome biogenesis (Ribi). Moreover, Runx1 deficient progenitor cells displayed a stress-resistant, pro-survival phenotype that has been suggested as an explanation for susceptibility to transformation [5]. In contrast, we observed no change in cell size or Ribi gene expression in Eμ-Myc lymphomas after deletion of Runx1, and a marked increase in stress sensitivity [3]. An obvious difference between our studies is the presence of constitutively active Myc, a major driver of Ribi. It is conceivable that loss of signalling to Myc is the key to reduced cell size in *Runx1^null^* progenitors, notwithstanding the observation that Runx1 can bind directly at ribosomal gene loci [5]. However, another potential explanation that must be considered is that functional redundancy within the gene family rescues Runx1 deficient Eμ-Myc cells. These cells express low levels of Runx3, which is modestly increased in the absence of Runx1 [our unpublished observations). It will be of great interest to explore the sensitivity of these cells to Runx3 knockdown and to recently developed allosteric inhibitors of RUNX-CBFβ binding [6].

While established Eμ-Myc lymphoma cells that express Runx1 have a clear selective advantage over excised cells, they are much less Runx-dependent than primary lymphomas *in vivo*. This is very encouraging with regard to the prospects for treating primary lymphomas, which are more likely to have intact p53. Whether loss of p53 is sufficient to explain the ability of Eμ-Myc cells to survive without Runx1 is as yet unclear. However, this finding highlights another relevant feature of the potent collaboration between Runx and Myc which appears to suppress p53 function in lymphoma cells *in vivo*, providing a paradigm for collaborating oncogenes that act synergistically by neutralising the cell's failsafe responses to oncogene over-activity [7]. One of the ‘grand challenges’ of contemporary cancer research is to find ways to target cancer cells over-expressing Myc. Inhibiting essential oncogenic cofactors such as the Runx family offers one potential solution. If the mechanism by which Myc and Runx combine to disable p53 also proves to be mediated by druggable targets, this special relationship may have a further pay-off.
